# Negative impact of low body mass index on liver cirrhosis patients with hepatocellular carcinoma

**DOI:** 10.1186/s12957-015-0713-4

**Published:** 2015-10-06

**Authors:** Qinggang Li, Hui Xing, Dan Liu, Hui Li

**Affiliations:** Department of Infectious Disease, First Affiliated Hospital of Harbin Medical University, No. 23 You Zheng Street, Harbin, 150001 People’s Republic of China; Department of Gastroenterology, Second Affiliated Hospital of Harbin Medical University, Xuefu Road, Harbin, 150086 People’s Republic of China

**Keywords:** Hepatocellular carcinoma, Body mass index, Prognosis, Mortality

## Abstract

**Background:**

The impact of obesity on the prognosis of hepatocellular carcinoma (HCC) has not been well characterized in a Chinese population. Therefore, the aim of this study was to examine the influence of BMI on the clinicopathological characteristics and mortality of patients with HCC.

**Methods:**

The study cohort consisted of 379 patients who were diagnosed with HCC at the First Affiliated Hospital of Harbin Medical University between June 2012 and August 2014. Study subjects were divided into two body mass index (BMI) categories: normal weight (BMI <23 kg/m^2^) and overweight (BMI ≥23 kg/m^2^).

**Results:**

Of the 379 patients, 44 (11.6 %) were underweight (<18.5 kg/m^2^), 172 (45.4 %) had a normal weight (18.5 ≤ BMI < 23.0), 133 (35.1 %) were overweight (23.0 ≤ BMI < 27.5), and 30 (7.9 %) were obese (BMI ≥27.5). After a median follow-up time of 296 (range, 15–720) days, 168 (44.3 %) patients died with median survival time of 159 (range, 15–690) days. Patients with lower BMIs also exhibited a higher liver-related mortality rate (60.6 vs. 22.7 %; *p* = 1.8 × 10^−13^) and a shorter survival time (353 days vs. 571 days; *p* = 6.2 × 10^−6^) than patients with higher BMIs. In multivariate analysis, the BMI class was also found to be a significant independent impact factor for overall survival (*p* = 2.2 × 10^−8^), age, alpha-fetoprotein level, Child–Pugh score, treatment strategy, antiviral treatment, extrahepatic metastasis, and tumor infiltration of the portal vein.

**Conclusions:**

Our data suggest that lower BMI has a significant impact regarding poor outcomes in patients with HCC. To better understand the impact of BMI on the prognosis of HCC patients, more large-scale cohort studies will be necessary.

## Background

Hepatocellular carcinoma (HCC) is a major global health burden and is the fifth most commonly diagnosed cancer in the world [1]. Its incidence and mortality have largely reflected the prevalence of hepatitis B (HBV) and C (HCV) viral infections, particularly in China. Recently, obesity has been found to be related to a variety of cancers including pancreatic cancer, colorectal cancer, and esophageal cancer [2–4]. Epidemiological data has shown that obesity not only increases the risk and progression of both HBV- and HCV-associated cirrhosis and HCC, but also is associated with non-B non-C HCC [5]. Moreover, the impact of obesity on the prognosis of HCC has not been well characterized in Chinese populations. Consequently, in the present study, we explored the significance of body mass index (BMI)-defined obesity on the survival of HCC patients in a Chinese population.

## Methods

### Patients

A total of 379 patients with liver cirrhosis diagnosed histologically or by typical clinical signs, diagnosed with HCC at the First Hospital of Harbin Medical University between June 2012 and August 2014, were studied retrospectively. A diagnosis of HCC was based on typical CT findings, namely hyperattenuation in the arterial phase and hypoattenuation in the equilibrium phase. HCC stages were classified according to the Barcelona Clinic Liver Cancer (BCLC) staging system [6]. Patients with a previous cancer history and those suspected of having metastatic hepatic cancer were excluded from our study. Written consent was obtained from each of our patients. The study was approved by the local ethics committee of the First Hospital of Harbin Medical University.

### Data collection

We recorded the following clinical data upon first admission and diagnosis in our department: age; sex; BMI; smoking and alcohol consumption history (ever or never); presence of diabetes mellitus; hepatitis infection status (HBV, HCV, HBV + HCV or none); Child–Pugh classification; BCLC stage; history of previous hepatitis treatment; and alpha-fetoprotein (AFP) level. Body weight was measured on admission. BMI was calculated as weight divided by height squared (kg/m^2^). According to the WHO classification for Asian populations, the patients were categorized as underweight (BMI <18.5), normal weight (18.5 ≤ BMI < 23.0), overweight (23.0 ≤ BMI < 27.5), and obese (BMI ≥27.5) [7]. Because the number of patients in the underweight group and the obese group was small, we divided all patients into two groups based on a BMI above or below 23.0 [8].

### Statistical analysis

All statistical analyses were carried out using SPSS version 17 (SPSS Inc. Chicago Illinois, USA) software. Differences in clinical features were assessed using the chi-square and Mann–Whitney *U* tests and ANOVA for continuous variables. A survival analysis was performed using the Kaplan–Meier procedure and compared with the log-rank test results. All *p* values were two-sided.

## Results

### Patient characteristics

Of the 379 eligible patients, 44 (11.6 %) were underweight (<18.5 kg/m^2^), 172 (45.4 %) had a normal weight (18.5 ≤ BMI < 23.0), 133 (35.1 %) were overweight (23.0 ≤ BMI < 27.5), and 30 (7.9 %) were obese (BMI ≥27.5). The median BMI of the entire cohort was 22.6 (14.8–34.1). Two hundred and sixteen (57.0 %) patients were included in the normal weight group (BMI <23.0) and 163 (43.0 %) in the overweight group (BMI ≥23.0). Baseline characteristics and clinicopathological features are detailed in Table 1.

**Table 1:** Baseline characteristics of study patients according to BMI

Characteristic	All cases	Normal weight (BMI <23 kg/m^2^)	Overweight (BMI ≥23 kg/m^2^)	*p* value
No. of patients	379 (100 %)	216 (57.0 %)	163 (43.0 %)	
Age, years	63.4 ± 11.7	64.0 ± 11.5	62.6 ± 11.8	0.199
Gender, male	219 (57.6 %)	116 (53.0 %)	103 (47.0 %)	0.064
Diabetes	71 (18.7 %)	35 (49.3 %)	36 (50.7 %)	0.147
Alcohol	95 (25.1 %)	54 (56.8 %)	41 (43.2 %)	0.973
Smoking	120 (31.7 %)	74 (61.7 %)	46 (38.3 %)	0.211
AFP ≥400 μg/L	228 (60.2 %)	71 (31.1 %)	157 (68.9 %)	0.760
Viral status				0.691
HBV	237 (62.5 %)	134 (56.5 %)	103 (43.5 %)	
HCV	125 (33.0 %)	70 (56 %)	55 (44 %)	
HBV + HCV	1 (0.3 %)	1 (100 %)	0 (0 %)	
None	16 (4.2 %)	11 (68.8 %)	5 (31.2 %)	
Previous treatment				0.717
Yes	113 (31.1 %)	66 (58.4 %)	47 (41.6 %)	
No	250 (68.9 %)	139 (55.6 %)	111 (44.4 %)	
Child–Pugh class				2.7 × 10^−4^
A	99 (26.1 %)	44 (44.4 %)	55 (55.6 %)	
B	107 (28.2 %)	57 (53.3 %)	50 (46.7 %)	
C	173 (45.6 %)	115 (66.5 %)	58 (33.5 %)	
Extrahepatic metastasis				0.113
Yes	36 (9.5 %)	25 (69.4 %)	11 (30.6 %)	
No	343 (90.5 %)	191 (55.7 %)	152 (44.3 %)	
Infiltration of portal vein				0.304
Yes	139 (36.7 %)	84 (60.4 %)	55 (39.6 %)	
No	240 (63.3 %)	132 (55.0 %)	108 (45.0 %)	
Treatment after diagnosis				1.3 × 10^−4^
Yes	151 (39.8 %)	68 (45.0 %)	83 (55.0 %)	
No	228 (60.2 %)	148 (64.9 %)	80 (35.1 %)	
BCLC stage				8.2 × 10^−5^
0 stage	10 (2.6 %)	3 (30.0 %)	7 (70.0 %)	
1 stage	64 (16.9 %)	28 (43.8 %)	36 (56.2 %)	
2 stage	78 (20.6 %)	38 (48.7 %)	40 (51.3 %)	
3 stage	54 (14.2 %)	32 (59.3 %)	22 (40.7 %)	
4 stage	173 (45.6 %)	115 (66.5 %)	58 (33.5 %)	

Mean patient age was 63.4 ± 11.7 years. HBV (62.5 %) infection was more prevalent than HCV in our cohort. After admission, 39.8 % of patients (151 cases) received surgery and/or transarterial chemoembolization treatment. The BCLC stage distribution was as follows: stage 0, 10 (12.6 %); stage A, 64 (16.9 %); stage B, 78 (20.6 %); stage C, 54 (14.2 %); and stage D, 173 (45.6 %). Other baseline characteristics are detailed in Table 1.

### Association of BMI with clinicopathologic variables

We examined the association between the categorical BMI and clinicopathologic variables (Table 1). In patients with advanced Child–Pugh scores and BCLC stages a trend towards lower weight was observed (*p* = 2.7 × 10^−4^ and *p* = 8.2 × 10^−5^, respectively). Of the 379 HCC patients, 52 (13.7 %) had undergone surgical therapy and 116 (30.6 %) had received TACE treatment, including 17 who had received surgery as well as TACE. As detailed in Table 1, HCC patients who had undergone curative treatment had a tendency towards higher BMIs (*p* = 1.3 × 10^−4^).

### Impact of BMI on the prognosis of patients with HCC

**Fig. 1:**
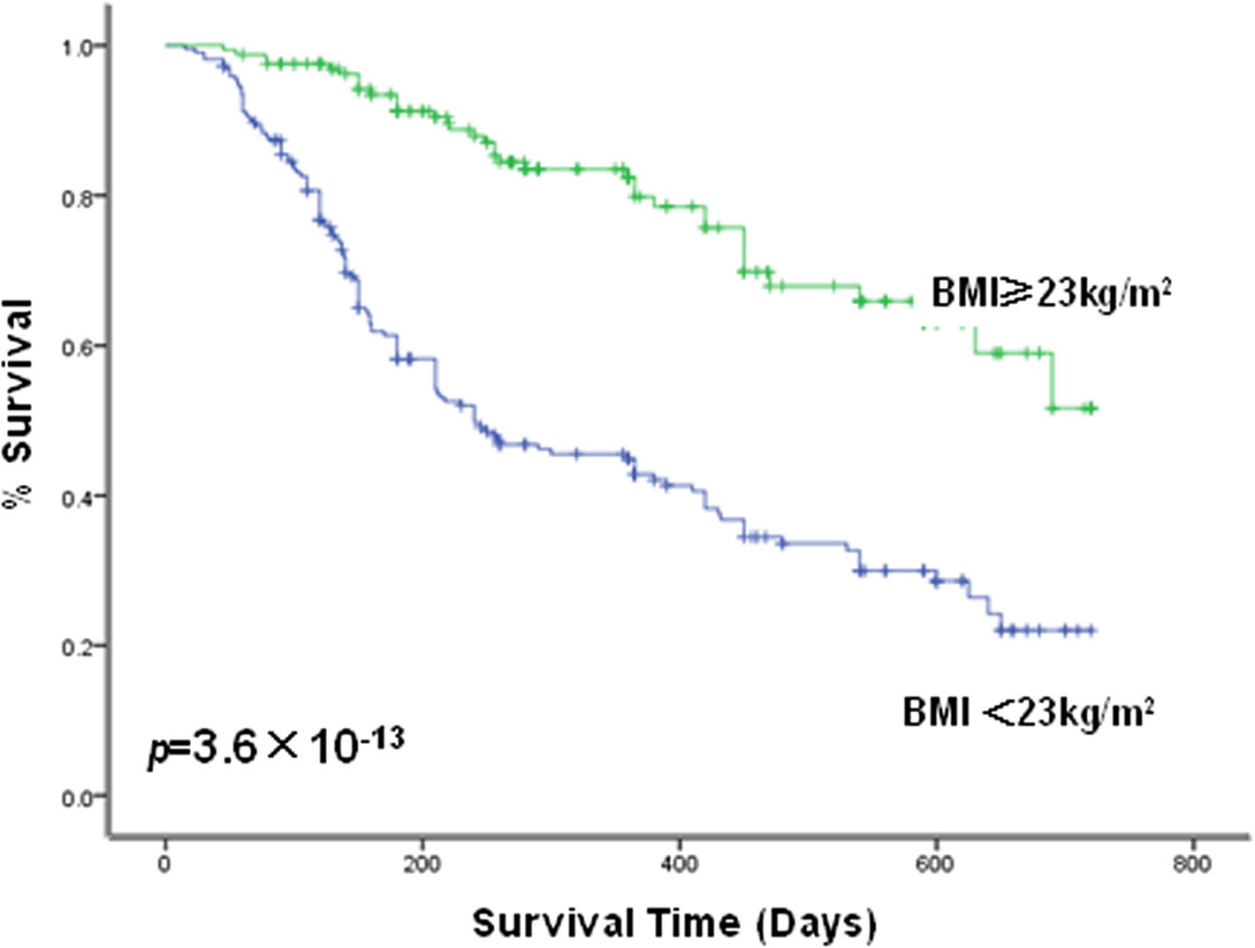
Relationship between BMI and prognosis of patients with HCC. Kaplan-Meier survival analysis demonstrating low BMI was significantly related to poor prognosis

After a median follow-up time of 296 (range, 15–720) days, 168 (44.3 %) patients had died with a median survival time of 159 (range, 15–690) days. Univariate analysis revealed that categorical Child–Pugh score and BCLC stage were significantly associated with overall-survival time. In particular, patients with higher Child–Pugh scores and BCLC stages had significantly shorter overall survival times as compared with lower-category patients (*p* = 3.6 × 10^−31^ and *p* = 1.4 × 10^−36^, respectively; Table 2). Similarly, median overall survival was shorter in patients with distant metastasis and infiltration of the portal vein (Table 2). Patients with HCC who received surgery and/or TACE treatment had a better clinical outcome (*p* = 2.5 × 10^−31^; Table 2). In addition, patients with a lower BMI also exhibited a higher liver-related mortality rate (60.6 vs. 22.7 %; *p* = 1.8 × 10^−13^; Table 2) and shorter survival time (353 vs. 571 days; *p* = 6.2 × 10^−6^) than patients with a higher BMI; this suggested that the BMI was a complementary predictor of poor prognosis in patients with HCC. As shown in Fig. 1, we found that patients with a higher BMI tended to have a longer overall survival (*p* = 3.6 × 10^−13^; Table 2).

**Table 2:** Overall survival for various characteristics based on Kaplan–Meier analysis

Variables	Overall survival (d) mean (95 % CI)	*p*
BMI		3.6 × 10^−13^
<23 kg/m^2^	353.9 (316.9–391.0)	
≥23 kg/m^2^	571.8 (532.3–611.4)	
Age		0.924
<60 years	444.9 (397.6–492.1)	
≥60 years	444.2 (406.8–481.5)	
Gender		0.537
Male	451.9 (413.7–490.2)	
Female	427.2 (382.8–471.6)	
Diabetes		0.563
Yes	440.6 (408.3–474.0)	
No	457.2 (388.0–526.5)	
Alcohol		0.453
Ever	425.8 (369.4–482.1)	
Never	451.4 (417.1–485.6)	
Smoking		0.085
Ever	411.6 (362.2–461.1)	
Never	462.1 (425.9–498.3)	
AFP		0.651
≥400 μg/L	456.8 (405.2–508.4)	
<400 μg/L	433.7 (396.3–471.1)	
Viral status		0.349
HBV	432.8 (396.0–469.6)	
HCV	459.8 (408.3–511.3)	
Previous treatment		0.502
Yes	459.8 (405.3–514.3)	
No	432.7 (396.9–468.5)	
Child–Pugh class		3.6 × 10^−31^
A class	627.1 (592.0–662.2)	
B class	479.7 (435.0–524.5)	
C class	232.0 (198.9–265.2)	
Extrahepatic metastasis		5.3 × 10^−13^
Yes	202.4 (148.4–256.4)	
No	471.2 (440.7–501.7)	
Infiltration of portal vein		1.4 × 10^−13^
Yes	259.9 (228.0–290.8)	
No	513.2 (479.2–547.1)	
Treatment after diagnosis		2.5 × 10^–31^
Yes	617.2 (589.2–645.2)	
No	259.7 (232.5–286.9)	
BCLC stage		1.4 × 10^−36^
0 stage	696.3 (652.7–739.8)	
1 stage	678.8 (653.2–704.4)	
2 stage	544.8 (500.2–589.4)	
3 stage	299.6 (257.9–341.2)	
4 stage	232.0 (198.9–265.2)

We performed multivariate analysis to identify factors that were independently associated with all-cause mortality. In multivariate analysis, BMI class was also found to be a significant independent impact factor regarding overall survival (*p* = 2.2 × 10^−8^), age, AFP, Child–Pugh score, treatment strategy, antiviral treatment, extrahepatic metastasis, and tumor infiltration of the portal vein (Table 3).

**Table 3:** Prognostic indicators of overall survival using multivariate analysis

Variables	Exp(B)	95 % confidence interval	*p*
Age	1.123	0.876–1.439	0.360
AFP	1.123	0.836–1.508	0.443
Child–Pugh stage	2.187	1.567–3.052	4.2 × 10^−6^
Treatment strategy	3.193	1.748–5.832	1.6 × 10^−4^
Portal invasion	0.772	0.535–1.112	0.164
BMI class	0.347	0.239–0.502	2.2 × 10^−8^
Extrahepatic metastasis	2.154	1.412–3.287	3.7 × 10^−4^
Antiviral treatment	1.330	0.942–1.878	0.105

## Discussion

Worldwide, the incidence of individuals with excessive body weight or obesity has increased markedly over the past decades. BMI has long been considered an important factor in predicting the prognostic outcome in patients with many types of cancer including colon, pancreatic, and esophageal. In HCC, the results of limited studies regarding the influence of obesity on treatment outcome are unclear. For instance, a high BMI is well established as a prognostic factor for poor survival. In a study that included 328 consecutive patients with primary HCC and 60 patients with recurrent HCC, patients in the obese group were reported to exhibit significantly poorer long-term prognosis than those in the non-obese group [9]. In contrast, a study by Okamura et al. reported that a low BMI was a significant prognostic factor for low overall survival after hepatectomy for HCC [10]. These discrepancies may have resulted from the difference in BMI category between the two study populations. In the latter study [10], the subjects were divided using a BMI of 18.5; patients with a low BMI of < 18.5 were commonly in a more vulnerable immunological state and had a higher frequency of more malignant HCC.

Several factors have been identified that influence the outcome of patients with HCC. Number and size of hepatic tumors, portal infiltration, metastasis, Child–Pugh score, and treatment algorithm are established impact factors regarding the survival of HCC patients [6, 11]. Malnutrition is a factor that has an important negative influence on the clinical performance status of patients with liver cirrhosis in addition to malignant disease. Merli et al. found that patients with impaired nutritional status did less well concerning liver transplantation [12]. In our study, we found that patients with a higher BMI had lower rates of liver-related mortality relative to patients with a lower BMI. Moreover, patients with a higher BMI showed a trend towards better overall survival in multivariate analysis. Therefore, BMI may be linked to the nutritional status of HCC patients and influence the prognostic outcome.

BMI is a simple measurement based on individual weight and height, and is widely used. However, some investigators have identified that calculation of the BMI is not suitable for the identification of malnourished patients with HCC, and that differences in body composition rather than in BMI may be a true determinant of prognosis. In this regard, Fujiwara et al. measured the skeletal muscle index, mean muscle attenuation, visceral adipose tissue index, subcutaneous adipose tissue index, and visceral-to-subcutaneous adipose tissue area ratios using CT rather than the BMI endpoint. They concluded that sarcopenia, intramuscular fat deposition, and visceral adiposity can independently predict mortality in patients with HCC [13]. However, these measurements are anthropometrically more difficult in clinical practice as compared with the BMI. In addition, nutritional status and body composition assessed using the BMI are relatively less precise in liver cirrhosis patients with HCC, in light of the fact that ascetic status could be overestimated in the calculation of BMI. However, malnutrition is the sole impact factor that affects the BMI, and ascites have only a limited effect on the BMI. Furthermore, ascites are one of the important components in the determination of the Child–Pugh score. Our study revealed clear evidence that a higher BMI is associated with better prognosis, although a high Child–Pugh score adversely affected HCC outcome. This suggests that BMI is an appropriate factor in the evaluation of nutritional status, despite its limitations.

Our study was the first to focus on BMI with regard to the prognosis of patients with HCC. However, it had some limitations. First, there was bias because it was a retrospective study. Second, CT data were not available for our patients, and only BMI was evaluated rather than body composition components.

## Conclusions

In conclusion, our data suggest that a lower BMI has a significant impact regarding the poor treatment outcomes of patients with HCC. To better understand the impact of BMI on the prognosis of HCC patients, more large-scale cohort studies will be required.
